# Moderate Beer Consumption Ameliorated Aging‐Related Metabolic Disorders Induced by D‐Galactose in Mice via Modulating Gut Microbiota Dysbiosis

**DOI:** 10.1002/fsn3.70678

**Published:** 2025-08-11

**Authors:** Xueyuan Fu, Changwei Wang, Zhaoxia Yang, Junhong Yu, Jianfeng Wang, Wanxiu Cao, Chuyi Liu, Hua Yin, Bafang Li, Xiaomei Feng, Fen Du, Hu Hou

**Affiliations:** ^1^ Qingdao Marine Biomedical Research Institute Qingdao China; ^2^ State Key Laboratory of Biological Fermentation Engineering of Beer Tsingtao Brewery Co., Ltd Qingdao China

**Keywords:** aging‐related metabolic disorders, d‐galactose, intestinal microbiota, moderate beer consumption

## Abstract

The process of aging is a multifaceted physiological phenomenon that entails the gradual deterioration of physical health. In recent years, fermented foods have garnered significant attention for their potential anti‐aging benefits; however, the effects of moderate consumption of beer on aging‐related metabolic disorders remain unexplored. Original beer, India Pale Ale (IPA), and Stout are widely consumed alcoholic beverages on a global scale. The objective of this study is to investigate and compare the effect of moderate consumption of these beers on aging‐related metabolic disorders in mice induced by D‐galactose (D‐gal), encompassing inflammation, organ impairment, oxidative stress, and dyslipidemia. Furthermore, potential mechanisms were elucidated through analysis of gut microbiota. Compared to the Original beer, oral administration of IPA and Stout effectively improved liver and kidney health in mice. All beers mitigated serum oxidative damage induced by D‐gal, with Stout exhibiting a more significant effect. Additionally, the Original beer was more effective at restoring intestinal microbiota diversity. Moreover, the tested beers elicited modifications in the composition of intestinal microbiota, including a decline in Firmicutes abundance and an elevation in Bacteroidota levels, accompanied by a decrease in harmful bacteria like Deferribacterota and an increase in beneficial bacteria like *Lactobacillus* and *Roseburia*. The research findings suggest that moderate consumption of Original beer, IPA, and Stout possesses the potential to mitigate D‐gal‐induced metabolic disorders through modulation of gut microbiota dysbiosis. Consequently, these results provide valuable insights into the favorable impacts associated with moderate beer consumption on human aging and health.

## Graphical abstract

The present study conducted a comparative analysis of the effects of moderate consumption of three prevalent beer types—Original beer, India Pale Ale (IPA), and Stout—on D‐galactose‐induced aging in mice. The findings demonstrate that the tested beers exhibit potential in mitigating metabolic disorders encompassing oxidative stress, inflammation levels, liver function, and blood lipid profiles. Furthermore, investigation into gut microbiota reveals that these beers enhance microbial diversity while promoting beneficial bacteria and suppressing harmful bacteria associated with aging, thereby exerting anti‐aging properties.

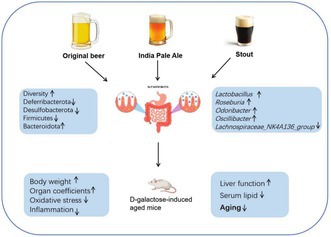

## 1 Introduction

The process of aging is closely associated with the onset and progression of age‐related ailments, including neurodegeneration, cardiovascular disorders, and diabetes (Liu et al. 2020). Increasing experimental evidence indicates that fermented food exhibits potential in ameliorating symptoms associated with age‐related metabolic disorders by mitigating neuroinflammation and oxidative stress. Furthermore, the involvement of fermenting microorganisms contributes significantly to their functional properties (Gates et al. 2022). Beer, brewed from hops, yeasts, and water, is the most widely consumed alcoholic beverage globally. The extracts of these ingredients, particularly hops, are rich in antioxidants, polyphenols, terpenes, flavones, and melanoidins that exhibit various antibacterial, anti‐inflammatory, and antioxidative effects (Chen et al. 2014). Based on many studies, the polyphenols present in wine or beer exhibit inhibitory effects on inflammatory causes and reactive oxygen radicals through the Nrf2 signaling pathway and are active on gut microbiota stimulation (Quesada‐Molina et al. 2019; Shahcheraghi et al. 2023; Zugravu et al. 2023). The therapeutic potential of natural flavones in age‐related diseases has been demonstrated (Zhang, Yan, et al. 2023). Melanoidins are normal constituents of beer, existing in varying proportions, and possess the potential to interact with microbiota through a prebiotic mechanism (Zugravu et al. 2023).

Original beer, India Pale Ale (IPA), and Stout are widely consumed alcoholic beverages on a global scale. The technological processes of malting and brewing, as well as subsequent storage, exert significant influence on the ultimate composition of beer (Anderson et al. 2019). Original beer is an unfiltered brew, devoid of filtration and high‐temperature treatment, thereby preserving the yeast's activity during fermentation as well as the authentic composition of malt and hops (Capece et al. 2018). The utilization of a substantial quantity of hops in the production of IPA imparts it with a distinctive hop aroma and an intensified bitterness profile. Moreover, the alcoholic content of IPA beer and malt wort typically exceeds that found in conventional beers. The Stout variety is characterized by its dark hue and is primarily brewed using roasted malt and rye malt, imparting a distinct toasted flavor profile (Kawa‐Rygielska et al. 2021).

Throughout centuries, the consumption of beer in moderation has been associated with numerous health benefits, including the prevention of diseases such as atherosclerosis (Marcos et al. 2021). However, its potential effects on aging‐related metabolic disorders have not yet been investigated. In this study, we conducted a comprehensive analysis of the biologically active ingredients in these types of beer. Additionally, we evaluated their alleviating aging effects using a D‐gal‐induced prematurely aged mouse model. The antioxidant and anti‐inflammatory capacities were assessed by measuring oxidative stress levels and inflammatory cytokines in the serum. Histopathological evaluation was performed to assess liver, kidney, and colon damage caused by D‐gal. Moreover, we examined the effects of tested beers on dyslipidemia induced by D‐gal. To elucidate the correlation between these effects and the composition of gut microbiota, we conducted a comparative analysis of intestinal microorganisms. Our study elucidates the distinct alleviating aging and health‐promoting effects of different beers in moderate consumption while further exploring their functional relationship with specific components and gut microbiota.

## 2 Materials and Methods

### 2.1 Materials

Original beer, IPA, and Stout were produced in China and supplied by State Key Laboratory of Biological Fermentation Engineering of Beer. The alcohol contents of the Original beer, IPA, and Stout were 5.42%, 5.83%, and 7.54%, respectively, and their bitterness values were 12.9 IBU, 36.6 IBU, and 31.6 IBU, respectively. According to the BJCP style guide, Original beer belongs to International Pale Lager, which is a highly‐attenuated pale lager without strong flavors, typically well‐balanced and highly carbonated. The IPA is English IPA, which is a bitter, moderately strong, very well‐attenuated pale British ale with a dry finish and a hoppy aroma and flavor. Stout belongs to Foreign Extra Stout, which is a very dark, rich, moderately strong, fairly dry stout with prominent roast flavors. The beers used were from the same batch by style. D‐galactose (D‐gal) was purchased from Beijing Solarbio Science and Technology Co. Ltd. (Beijing, China). Commercial kits purchased from Nanjing Jiancheng Institute of Biotechnology (Nanjing, China) were used for determining superoxide dismutase (SOD), catalase (CAT), glutathione peroxidase (GSH‐Px), malondialdehyde (MDA), alanine aminotransferase (ALT), aspartate aminotransferase (AST) and alkaline phosphatase (AKP). Elisa assay kits interleukin 6 (IL‐6), interleukin 15 (IL‐15), tumor necrosis factor‐α (TNF‐α), low density lipoprotein total (LDL), total cholesterol (TC), and triglyceride (TG) were purchased from Jiangsu Jingmei Institute of Biotechnology (Nanjing, China).

### 2.2 Evaluation of the Total Phenol Content (TPC)

The TPC in beer was determined using the Folin–Ciocalteu (FC) method (Velickovic et al. 2020). Aliquots of the sample (0.2 mL) were mixed with 2.5 mL of FC reagent and supplemented with 2.5 mL of 7.5% sodium carbonate (Na_2_CO_3_). After incubation at 40°C for 1 h, absorbance was measured at 760 nm against a blank solution. Results were calculated based on a gallic acid (GA) calibration curve.

### 2.3 Evaluation of the Total Flavonoid Content (TFC)

The TFC was determined using the spectroscopic technique (Velickovic et al. 2020). A mixture of 4.1 mL 80% ethanol (C_2_H_5_OH), 0.1 mL Al (NO_3_)_3_ and 0.1 mL 1 M potassium acetate (CH_3_COOK) was added to 1 mL aliquots of sample or ethanol (blank). After incubation for 40 min, the absorbance at 510 nm was measured. The TFC values were calculated based on the rutin calibration curve.

### 2.4 Evaluation of the Melanoidins

The isolation of melanoidins was conducted following the methods described by Haifeng Zhao et al. (2013). Specifically, 30 mL of beer was placed in dialysis tubing with a molecular weight cut‐off at 5000 and then dialyzed in 1000 mL of water for 48 h at 4°C with stirring. The retentates were freeze‐dried and subsequently utilized for determining the melanoidin content in different beers. The levels of melanoidins were determined by quantifying the weight of the yellow‐brown powders obtained.

### 2.5 Animal Experiments

We acquired male ICR mice, aged 8 weeks and specific‐pathogen‐free (SPF), from Vital River Laboratory Animal Technology in Beijing, China. The mice were maintained under controlled conditions at a temperature of 23°C ± 2°C and randomly divided into 5 groups (*n* = 10). The mice in the normal (N) group did not receive any injections, while the other groups received daily subcutaneous injections of D‐gal (25 mg kg^−1^) for a period of 8 weeks to induce an aging model. The N and aging model (M) groups were treated with saline by gavage, whereas the Original beer group (O), IPA group (I) and Stout group (S) received a daily oral gavage of 0.5 mL beer (This is equivalent to an adult drinking 100 mL of beer every day) for a duration of 4 weeks, respectively.

The animals' body weight was recorded weekly. Fresh fecal samples were collected and immediately preserved at −80°C until assayed. Subsequently, mice were sacrificed; the blood was collected. The livers, spleens, and kidneys of the mice were excised and weighed, and their weights relative to the final body weight (organ coefficient) were calculated using the following formula: organ coefficient (mg/g) = organ weight (mg)/body weight (g) (Sun et al. 2023). Histological analysis was performed on sections of the liver, kidney, and colon tissues, which were fixed in 4% paraformaldehyde. This study was approved by the Scientific Ethics Committee of Qingdao Marine Biomedical Research Institute; the ethical approval code is E‐MBWM‐2024‐23.

### 2.6 Biochemical Assays

The mice blood was centrifuged at 4500 r/min for 10 min at 4°C to isolate serum. Assay kits were employed to assess the levels of SOD, CAT, GSH‐Px, MDA, ALT, AST, and AKP in the isolated serum. The levels of inflammatory cytokines IL‐6, IL‐15, and TNF‐α, as well as blood lipid indexes LDL, TC, and TG in serum were quantified using ELISA assay kits following the manufacturers' instructions.

### 2.7 Histopathological Evaluation

The liver, kidney, and colon tissues fixed with paraformaldehyde were sectioned into 3 μm slices and subsequently embedded in paraffin. Following repeated dewaxing with xylene, the sections were rehydrated using alcohol and stained with hematoxylin and eosin (H&E). Finally, their morphology was observed under an Olympus CX31RTSF microscope (OLYMPUS Corporation, Japan) to assess histopathological changes in the liver, kidney, and colon tissues.

### 2.8 Microbial Diversity Analysis of Colonic Contents

The gut microbiota composition of mice feces was determined by 16S rRNA gene amplification. Total DNA from the colonic contents was extracted using a DNeasy PowerSoil kit (Qiagen, Hilden, Germany). The PCR products were purified with Agencourt AMPure XP beads (Beckman Coulter Co., USA) and quantified using Qubit dsDNA assay kit. Sequencing was performed on an Illumina NovaSeq6000 with two paired‐end read cycles of 250 bases each (Illumina Inc., San Diego, CA; OE Biotech Company; Shanghai, China). Raw sequencing data were in FASTQ format. The representative read of each Amplicon Sequence Variant (ASV) was selected using QIIME 2 package.

Functional inference associated with aging was determined by identifying Kyoto Encyclopedia of Gene and Genomes (KEGG) pathways. The R packages (v.4.3.1) used in correlation analysis were Hmisc, PerformanceAnalytics, psych, pheatmap, and reshape2. Network analysis was conducted using the “WGCNA” R package (v.4.3.1). The microbial network interactions were visualized using Cytoscape (version 3.10.0).

### 2.9 Statistical Analysis

The experimental results were reported as mean ± standard deviation (SD) and analyzed using SPSS V22.0 (SPSS Inc., Chicago, IL, USA). To determine significant differences between treatment groups, one‐way ANOVA was performed with a significance level of *p* < 0.05.

## 3 Results and Discussion

### 3.1 Concentrations of TPC, TFC, Melanoidins Content in Tested Beers

As shown in Table 1, the TPC content ranged from 218.13 to 269.13 mg/L, with the Original beer exhibiting the lowest content, while IPA and Stout demonstrated significantly elevated levels compared to Original beer (*p* < 0.05); however, there was no significant difference between IPA and Stout. The TFC in Original beer and Stout was 139.44 and 144.59 mg/L, respectively, which exhibited a significant increase compared to IPA (*p* < 0.05). Both IPA and Stout displayed higher levels of melanoidins compared to Original beer. In conclusion, Stout demonstrated higher levels of TPC, TFC, and melanoidin content when compared to Original beer and IPA.

**TABLE 1:** Concentrations of TPC, TFC, melanoidins in tested beers.

Beer type	TPC (mg/L)	TFC (mg/L)	Melanoidins (mg/L)
Original beer	218.13 ± 0.95^a^	139.44 ± 2.82^a^	288.03 ± 3.34^a^
India Pale Ale	260.43 ± 0.83^b^	111.37 ± 2.11^b^	293.84 ± 1.82^a^
Stout	269.13 ± 0.73^b^	144.59 ± 3.99^a^	296.68 ± 0.48^a^

*Note:* The values were reported as mean ± SD (*n* = 3). Mean values with different superscript letters (a, b) within the same column indicate statistical significance (*p* < 0.05).

Beer styles exhibit variations in their composition, wherein the selection and quantity of malts, hops, as well as the technological processes serve as determining factors for the final product's components. One analysis demonstrated that approximately 30% of the polyphenols present in beer are derived from hops, while the remaining 70%–80% can be attributed to malt (Arranz et al. 2012). The higher polyphenol content of IPA can be attributed to the increased addition of hops during fermentation. Stout exhibited the highest flavonoid content among all the beers tested. The chemical composition of rye exhibits a higher concentration of flavonoids and a diverse range of polyphenols, including phenolic acids, in comparison to commonly consumed wheat (Angioloni and Collar 2011; Gumul and Berski 2021). The use of rye as the primary ingredient in Stout may contribute to its higher levels of polyphenols and flavonoids. It was proved that significant increases in melanoidins were observed during the roasting of barley (Aljahdali et al. 2020). Stout exhibits the highest melanoidins content, which can be attributed to the utilization of baked or rye malt as primary fermentation ingredients in the brewing process.

### 3.2 Body Weight and Organ Coefficient in Mice

**FIGURE 1:**
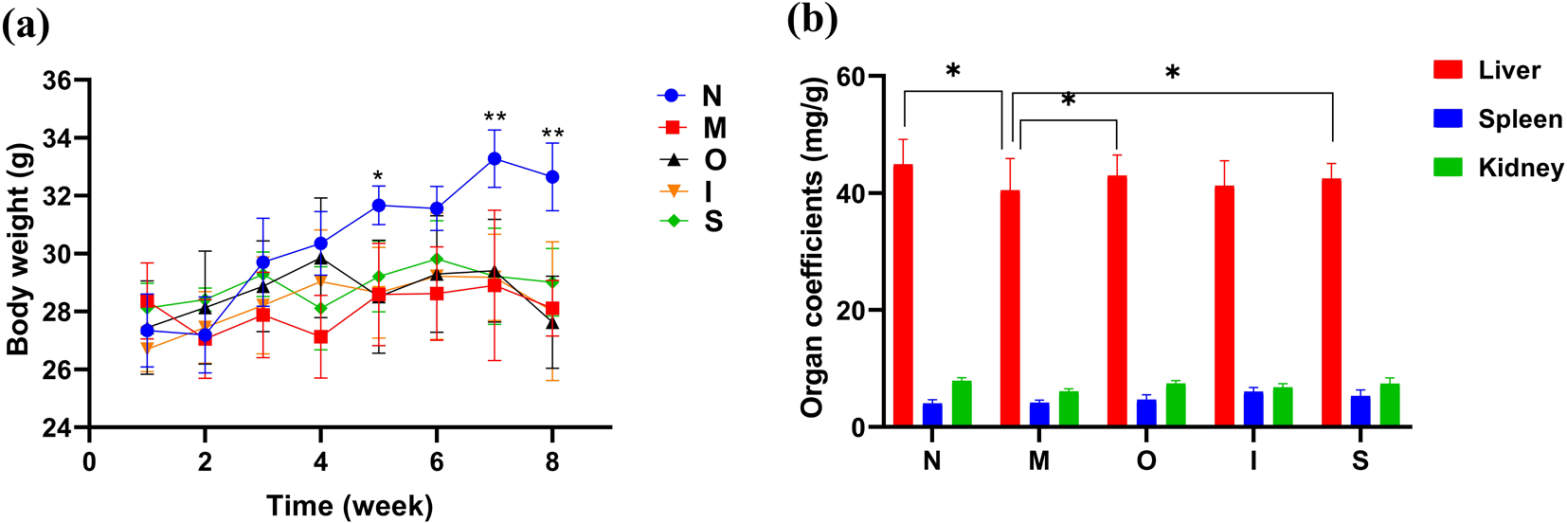
Body weight and organ coefficients in mice. (a) Body weight measurement; (b) Liver, spleen, kidney coefficients. *p < 0.05, **p < 0.01. Data are expressed as mean ± SD (*n* = 10).

The body weight and organ coefficients can indeed serve as a proxy for mouse growth status to some extent. As depicted in Figure 1a, the mice treated with D‐gal exhibited a significant decline compared with normal mice in body weight starting from the fourth week (*p* < 0.05). However, the tested beers were able to counteract this reduction in body weight; among them, Stout demonstrated superior efficacy in reversing this effect. As depicted in Figure 1b, treatment with D‐gal resulted in a reduction of liver and kidney coefficients, whereas administration of Original beer and Stout exhibited an obvious reversal of the decline in liver coefficients (*p* < 0.05). A previous study has shown that moderate beer consumption does not cause liver damage in Wistar rats (Caon et al. 2021). Furthermore, all three types of beers demonstrated restoration in kidney and spleen coefficients compared to the M group.

Aging is accompanied by a gradual decline in physical function; this decline ultimately leads to a loss of tissue and organ function (He et al. 2024). Excessive administration of D‐gal in mice results in increased production of reactive oxygen species (ROS) and significantly exacerbates tissue damage caused by oxidative stress (Xiong et al. 2023). The findings in this study suggest that moderate consumption of Original beer, IPA, and Stout may have potential efficacy in enhancing organ health among aging mice.

### 3.3 Oxidative Stress and Inflammation Levels in Mice

**FIGURE 2:**
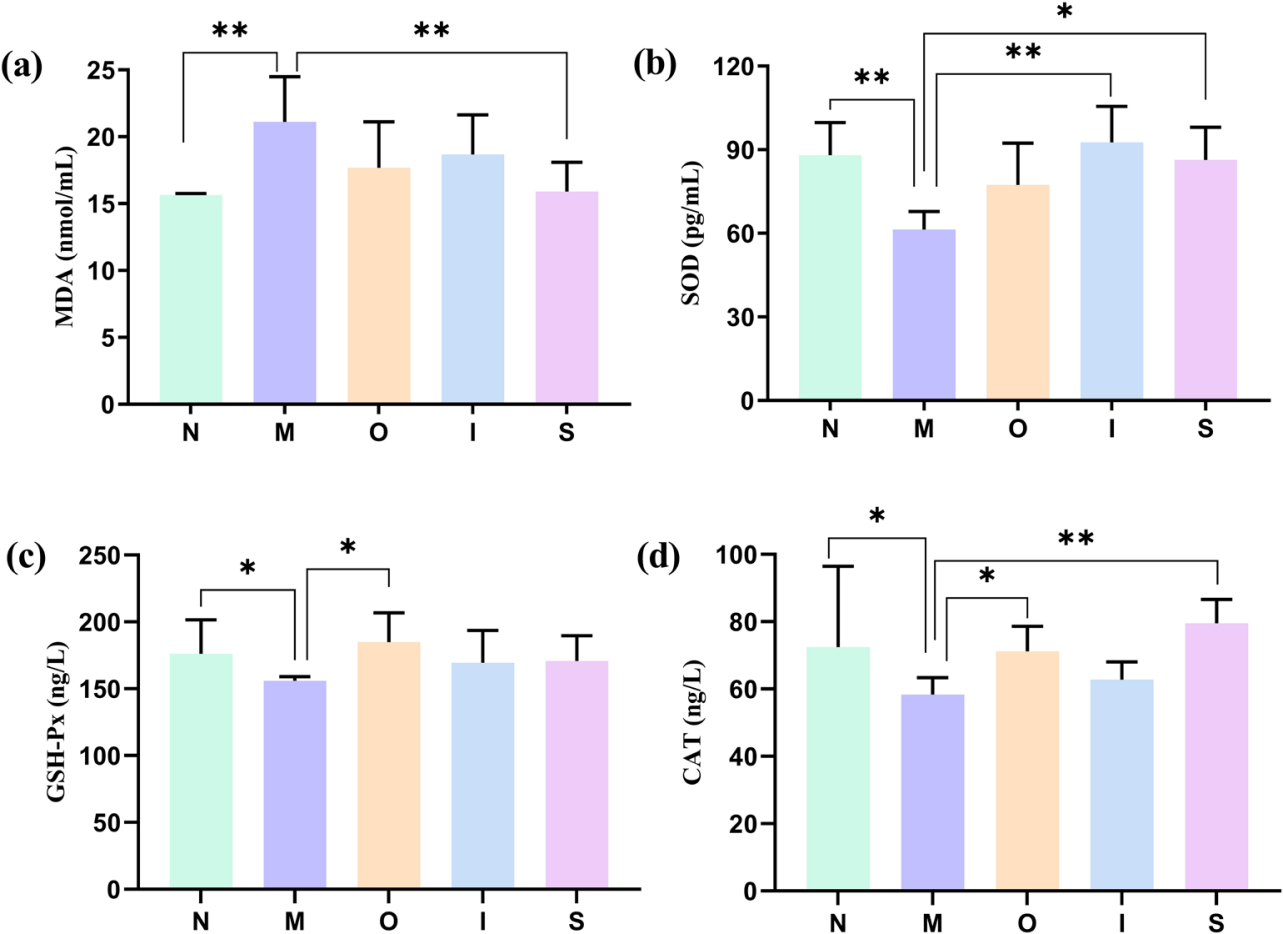
Oxidative stress levels in mice. (a) MDA, (b) SOD, (c) GSH‐Px, (d) CAT levels in serum. *p < 0.05, **p < 0.01. Data are expressed as mean ± SD (*n* = 10).

As depicted in Figure 2, compared to the normal group, D‐gal induction resulted in a significant reduction in the SOD concentration (Figure 2b), GSH‐Px level (Figure 2c), and CAT activity (Figure 2d) (*p* < 0.01, *p* < 0.05, *p* < 0.05 respectively), accompanied by a significant increase in MDA level (Figure 2a) (*p* < 0.01). These findings suggest the occurrence of oxidative stress injury in D‐gal‐treated mice. Original beer treatment exhibited remarkable improvements on antioxidant enzymes GSH‐Px and CAT, while IPA intervention effectively reversed SOD level (*p* < 0.01). Stout treatment demonstrated substantial enhancements on SOD and CAT activities as well as a decrease in MDA level. These findings suggest that supplementation with the tested beers effectively alleviates the redox imbalance induced by D‐gal, with Stout showing more pronounced improvement effects.

Previous study has indicated that the main antioxidant compounds in beer are phenolic and melanoidins (Borsa et al. 2022). Flavonoids are a type of polyphenol. The presence of Maillard components has been attributed to the positive correlations observed between antioxidant activity and malt color in several studies. Furthermore, a direct association was identified between melanoidin content in beers and their antioxidant capacity (Xiong et al. 2023). The high in vivo antioxidant activity of Stout may be attributed to the utilization of specialized malts, such as rye malts.

**FIGURE 3:**
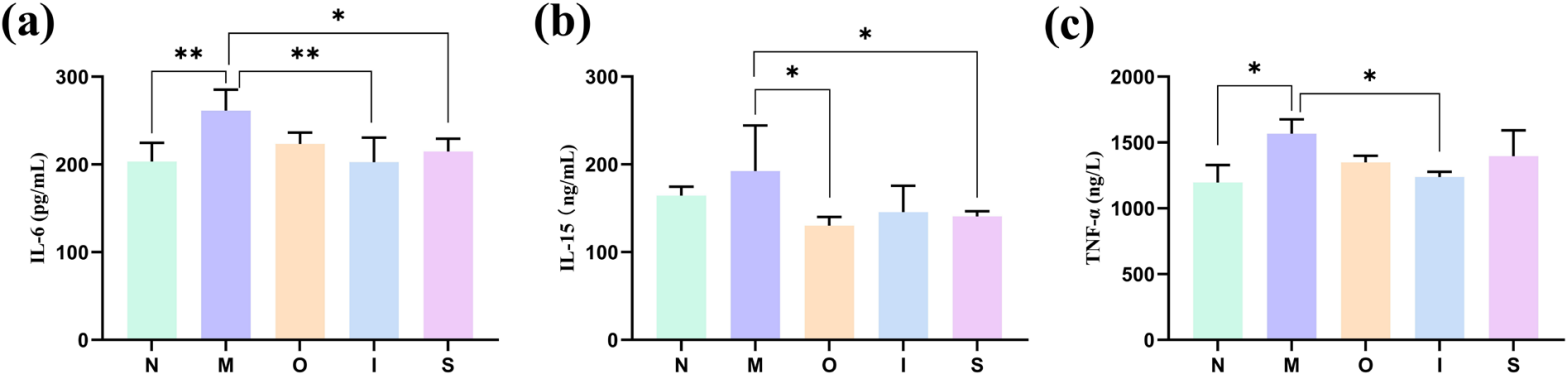
Inflammatory factors levels in mice. (a) IL‐6, (b) IL‐15, (c) TNF‐α levels in serum. *p < 0.05, **p < 0.01. Data are expressed as mean ± SD (*n = 10*).

Proinflammatory factors such as IL‐6, IL‐15, and TNF‐α significantly contribute to inflammation‐related aging as well as various age‐related diseases. IL‐6 is commonly referred to as the “cytokine of gerontologists” (Xu et al. 2019). As depicted in Figure 3, the levels of IL‐6 (Figure 3a) and TNF‐α (Figure 3c) exhibited a substantial increase in the M group. However, both IPA and Stout effectively reduced the level of IL‐6. Treatment with Original beer and Stout significantly decreased the level of IL‐15 compared to the M group (Figure 3b) (*p* < 0.05). Moreover, IPA demonstrated a noticeable decrease in TNF‐α level compared to the M group (*p* < 0.05). In conclusion, both IPA and Stout exhibited superior anti‐inflammatory properties when compared to Original beer. The process of inflammation is linked to oxidative stress and the aging process. Chronic inflammation can exacerbate telomere dysfunction by increasing DNA damage mediated by ROS, which accelerates the accumulation of senescent cells. Conversely, aging can intensify chronic inflammation and ROS production, creating a detrimental cycle that impedes tissue regeneration and expedites senescence (Jurk et al. 2014). Therefore, one mechanism for the anti‐inflammatory effect of tested beers of the aging mice may be ascribed to their antioxidant activitie Previous study proved that kaempferol, ferulic acid, xanthohumol/isoxanthohumol in beer are important beer compounds of anti‐inflammatory/anti‐oxidative effects (Chen et al. 2014). The iso‐α‐acids present in beer exhibit neuroprotective effects by suppressing neuroinflammation and enhancing cognitive function (Ano et al. 2017).

### 3.4 Hepatic Function and Blood Lipid Levels in Mice

**FIGURE 4:**
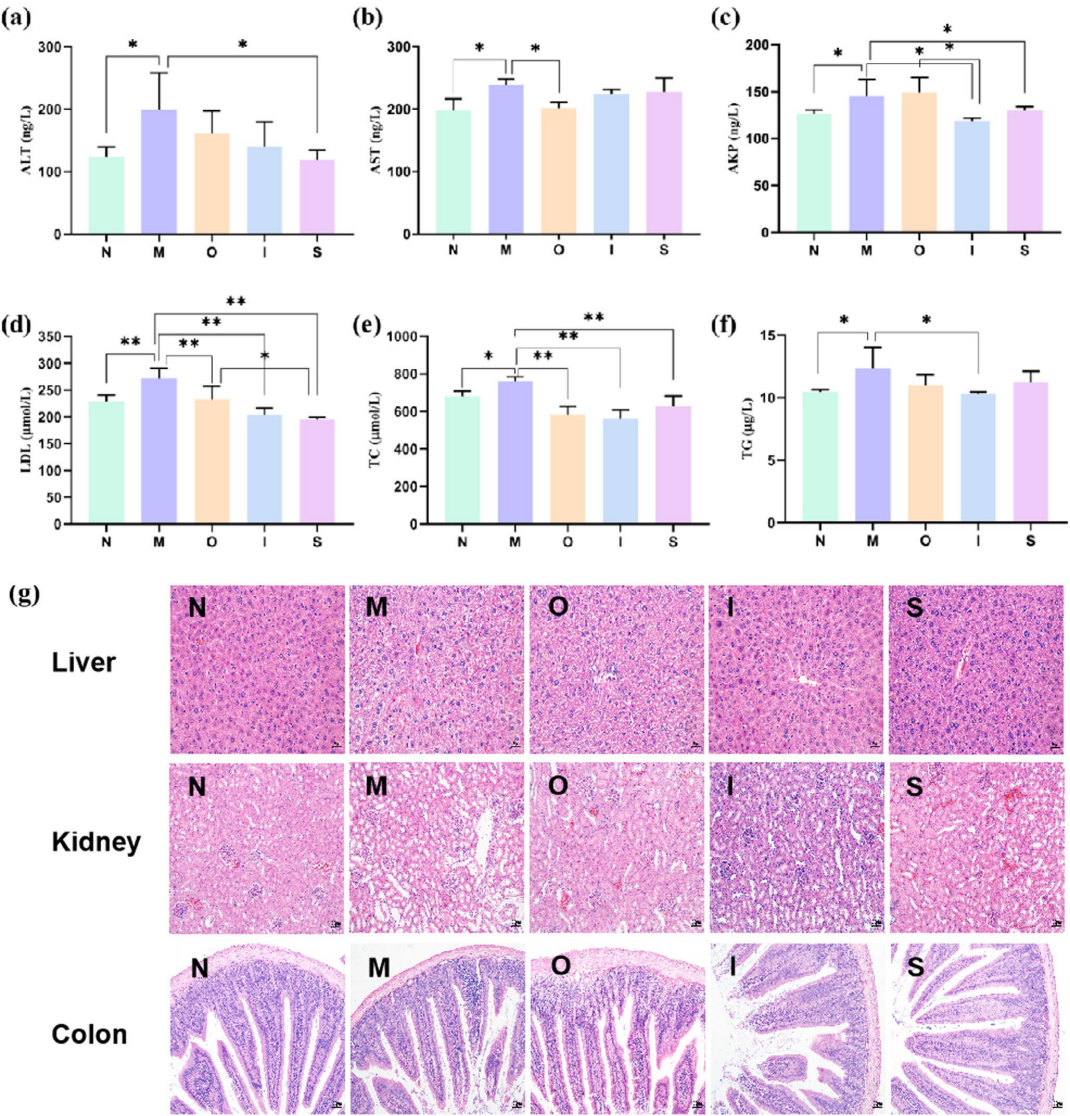
*Hepatic fun*ction, blood lipid levels, and organ morphology in mice. (a) ALT, (b) AST, (c) AKP, (d) LDL, (e) TC, and (f) TG levels in serum; (g) The liver, kidney, colon histopathological analysis stained with H&E, magnification × 200. *p < 0.05, **p < 0.01. Data are expressed as mean ± SD (*n = 10*).

The levels of serum ALT, AST, and AKP serve as crucial and sensitive biochemical indicators for evaluating liver function, with their aberrant elevation potentially leading to hepatic injury and necrosis (Xu et al. 2018). As shown in Figure 4, the D‐gal‐treated mice exhibited a significant elevation in ALT, AST, and AKP values compared to the normal mice (*p* < 0.05). However, administration of Stout resulted in a noteworthy reduction in serum ALT and AKP levels (*p* < 0.05). IPA demonstrated a significant decrease in AKP compared to the M group (*p* < 0.05), while Original beer was found to significantly decrease AST levels compared to the M group (*p* < 0.05). As shown in Figure 4g, liver cells exhibit intact structural features with clear cell boundaries and complete nuclei in the normal group. In contrast, liver cells of aging mice induced by D‐gal showed evident structural disarray, characterized by ballooning degeneration, increased cellular necrosis, and unclear cell boundaries. Meanwhile, oral administration of Original beer showed slight improvement in liver damage, manifested as reduced ballooning degeneration. The IPA and Stout treatment groups demonstrated significant protective effects similar to the normal group. These findings suggest that the tested beers effectively alleviate D‐gal‐induced liver cell injury, with IPA and Stout exhibiting more pronounced protective effects. It has been indicated that drinking beer in low doses will not damage the liver (Caon et al. 2021). A previous study has proved that some polyphenol compounds like chlorogenic acid were able to protect D‐gal‐induced liver damage (Feng et al. 2016). Hop‐derived humulinones were proved to reveal protective effects in in vitro models of hepatic steatosis, inflammation, and fibrosis (Mahli et al. 2023). Therefore, this study provides evidence to support the notion that moderate beer consumption does not induce alcohol‐related hepatic damage, and its hepatoprotective effect may be contingent upon the bioactive constituents it encompasses.

The aging process is frequently accompanied by dyslipidemia. In this study, we assessed the serum levels of LDL, TC, and TG (Figure 4d–f). The D‐gal‐treated mouse model exhibited significantly elevated levels of LDL, TC, and TG compared to the normal group (*p* < 0.01, *p* < 0.05, *p* < 0.05). Remarkably, both Original beer and Stout were able to effectively reverse the increased levels of LDL and TC (*p* < 0.01). Additionally, IPA demonstrated notable efficacy in reducing LDL, TC, and TG levels (*p* < 0.01, *p* < 0.01, *p* < 0.05). Collectively, these results provide compelling evidence that the tested beers possess considerable potential in ameliorating D‐gal‐induced dyslipidemia, with IPA demonstrating superior efficacy. The efficacy of hop cones in treating metabolic syndrome, particularly through lipid metabolism, has been substantiated by the demonstrated effectiveness of polyphenols and bitter acids (iso‐α‐acids) (Dostálek et al. 2017). Relevant to the lipid profile of postmenopausal women, moderate beer consumption exhibits a beneficial impact (Trius‐Soler et al. 2023). According to a publication, the administration of either alcoholic solution (4%) or lyophilized beer exhibited significant favorable effects on plasma lipidemic and antioxidant markers (Ambra et al. 2021). In conclusion, the presence of numerous functional ingredients in the tested beers contributes to their hepatoprotective effects and enhancement of lipid metabolism in D‐gal‐treated mice.

### 3.5 Kidney and Colon Morphology in Mice

As shown in Figure 4g, the renal cortex of mice in the M group exhibited significantly thickening of the mesangial matrix and the appearance of vacuolar degeneration compared with the normal mice. In addition, some interstitial areas showed inflammatory cell infiltration, implicating the inflammation of D‐gal‐treated mice. However, compared with the M group, the mice receiving various beers had relatively intact glomerular structure in the renal cortex, along with significantly reduced vacuolar degeneration and interstitial inflammatory cell infiltration. The results of the H&E staining demonstrated no discernible alterations in the structural integrity of the colon across all five experimental groups. The results suggest that tested beers may possess certain improvement effects on the pathological changes of kidney injury induced by D‐gal.

### 3.6 Diversity of the Intestinal Microbiota in Mice

**FIGURE 5:**
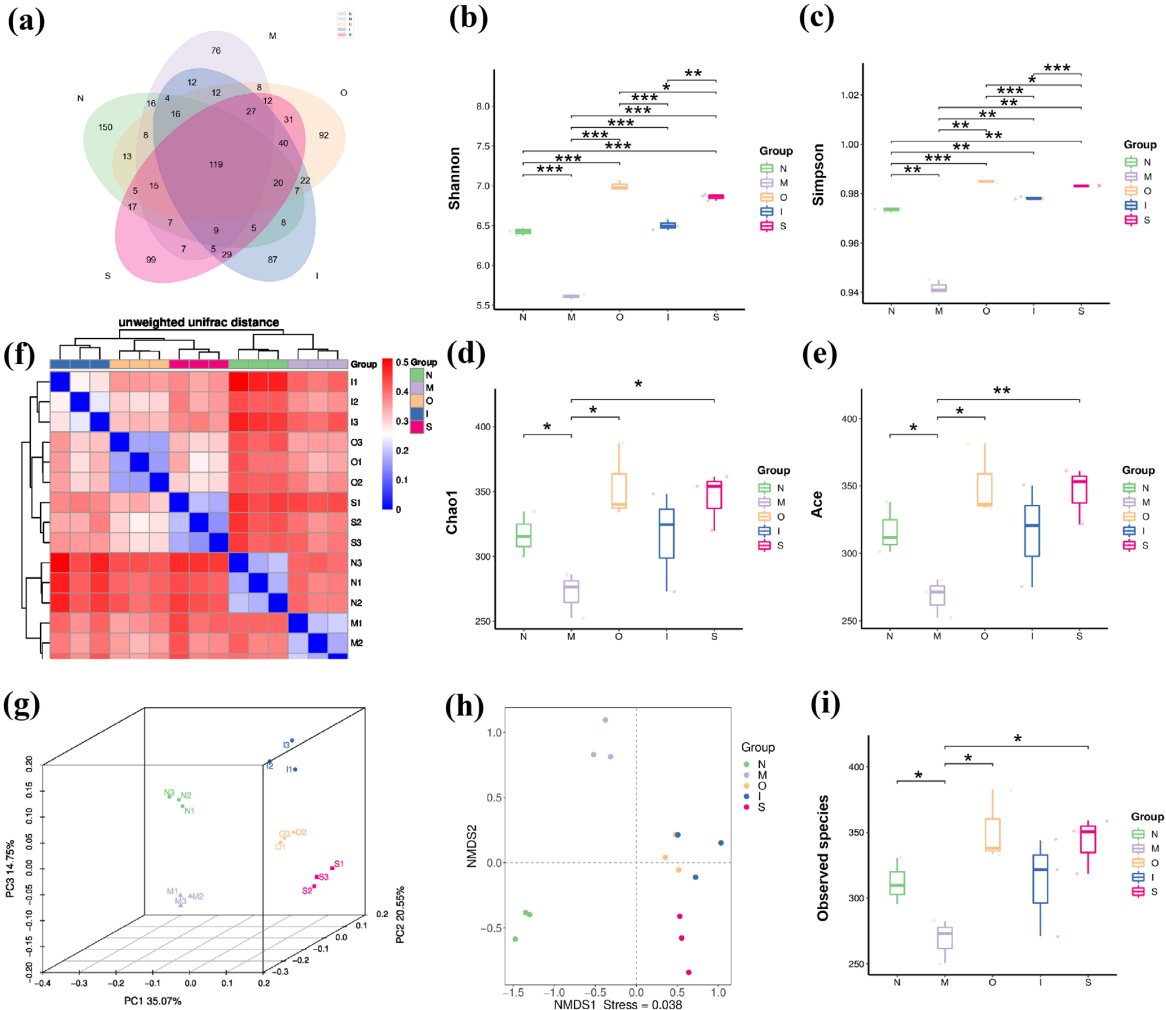
*Diversity of the in*testinal microbiota in mice. (a) Venn diagram for each test group; (b) Shannon, (c) Simpson, (d) Chao1, (e) ACE indices; (f) Beta diversity analysis of intestinal microbiota using the unweighted UniFrac diversity distance, (g) PCoA analysis, (h) NMDS analysis; (i) The observed species number. *p < 0.05, **p < 0.01, ***p < 0.001. Data are expressed as mean ± SD (*n* = 3).

Numerous studies have consistently demonstrated that aging is associated with a decline in the diversity of gut microbiota (Choi et al. 2018). Venn diagrams were used to visualize the similarity and differentiation of all the samples; the study found a total of 978 amplified sequence variants (ASVs) in all the groups (Figure 5a). The total number of ASVs in N, M, O, I S groups were 419, 353, 447, 422, and 447, respectively. The observed species in the Original beer and Stout treatment groups exhibited significantly higher levels compared to those of the aging model group, as depicted in Figure 5i (*p* < 0.05). The alpha‐diversity was assessed to ascertain alterations in the microbial species composition among different groups of mice, as shown in Figure 5b–e. The M group exhibited a significant decrease in all 4 indices when compared with the N group (*p* < 0.05); the O and S groups displayed a significant increase in all indices compared with the M group (*p* < 0.05), while the I group could obviously increase the Shannon and Simpson indices (*p* < 0.01).

Beta diversity analysis, aided by unweighted UniFrac distance analysis, Principal Coordinate Analysis (PCoA) and Non‐metric Multidimensional Scaling (NMDS) techniques, provides valuable insights into the comparison of intestinal microflora composition among various groups. The results showed that there were significant differences in beta diversity among the groups (Figure 5f). The PCoA and NMDS analysis clearly demonstrate the five groups occupy distinct regions of the coordinate axis, indicating significant differences in their microbiota compositions (Figure 5g,h). In conclusion, these findings suggest that three types of beers can enhance the decreased microbial diversity and richness induced by D‐gal, with Original beer demonstrating superior advantages compared to IPA and Stout. The absence of filtration and high temperature treatment in Original beer results in its richness in *Saccharomyces cerevisiae* strain, which may modulate gut microbiota and exert beneficial effects on intestinal microbial diversity. The reported findings indicate that treatments involving beer and beer/yeast significantly augmented the richness of gut bacterial population in 3xTg‐AD mice, thereby rendering their microbiota more akin to that of healthy individuals (Cecarini et al. 2022). Previous work has proved that moderate consumption of beer has a positive effect on human health by enrichment of the gut microbiota diversity (Hernández‐Quiroz et al. 2020); this is consistent with our research findings.

### 3.7 Community Structure of the Intestinal Microbiota in Mice

**FIGURE 6:**
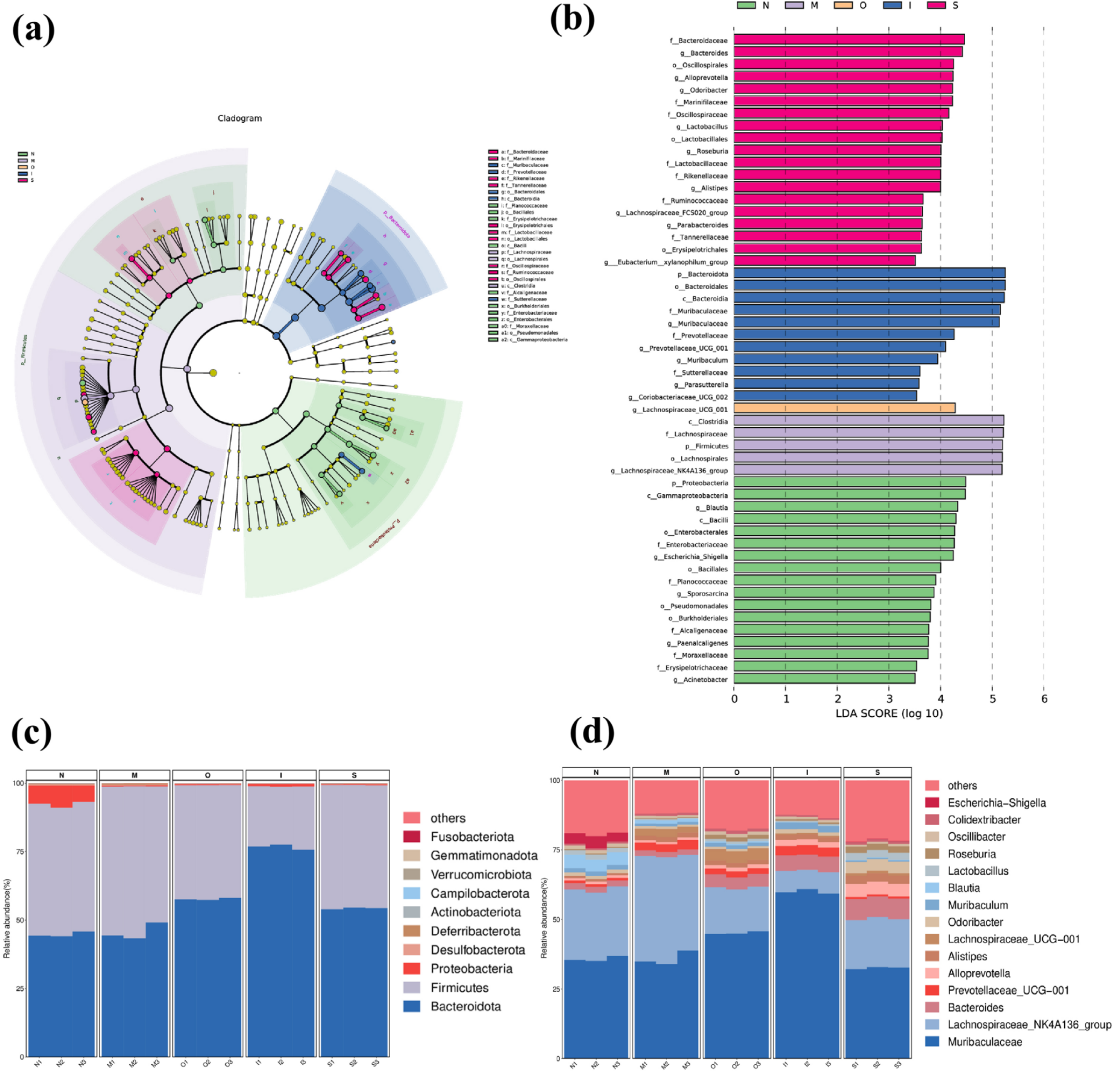
Linear discriminant analysis and gut microbial composition. (a) Cladogram illustrating highly abundant taxa across various treatments; (b) Taxonomic cladogram obtained from LEfSe analysis; (c) Gut microbial composition at the phylum level; (d) Gut microbial composition at the genus level.

**FIGURE 7:**
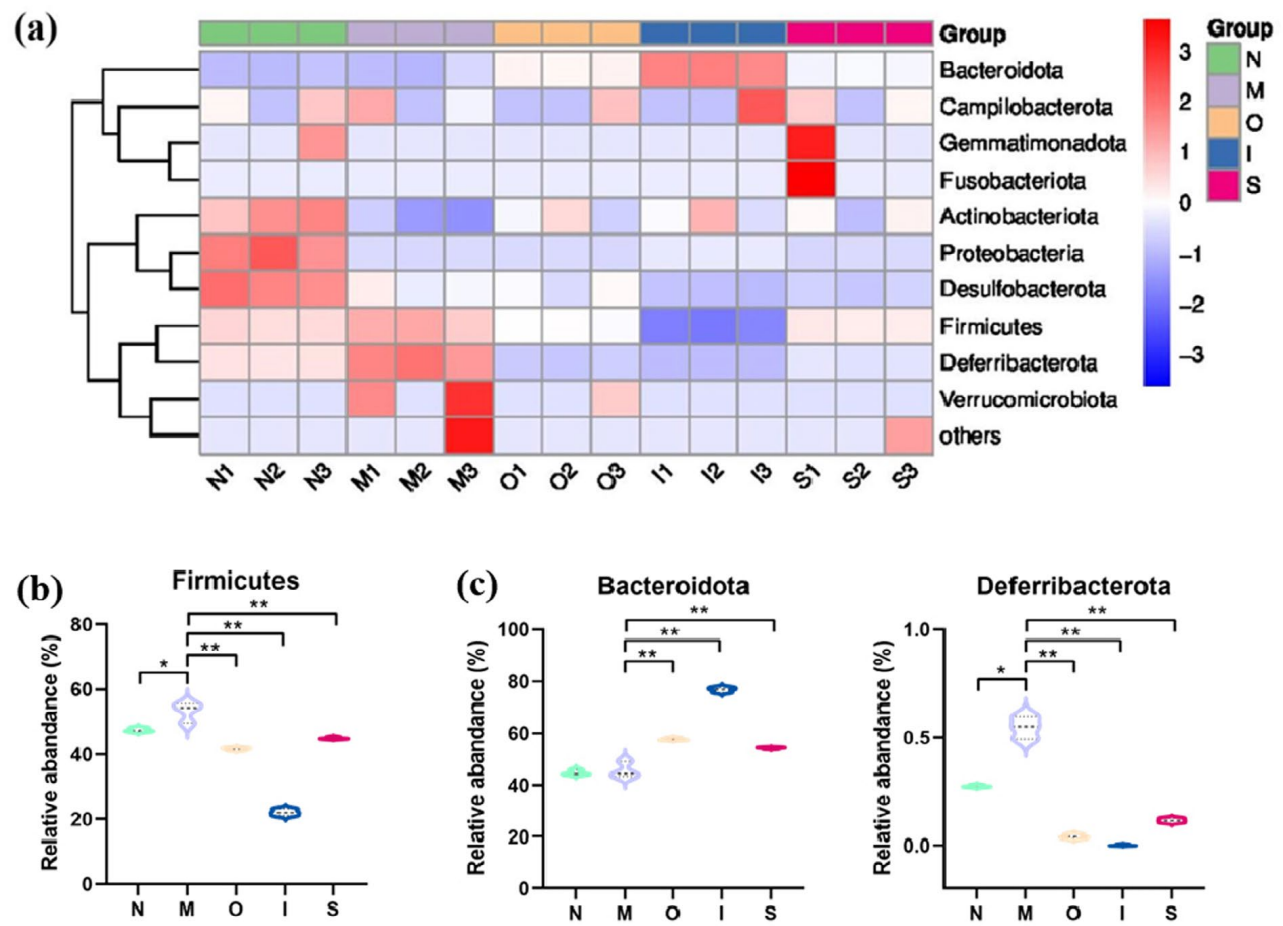
Gut microbial analysis at the phylum level. (a) The heatmap analysis at the phylum level; (b) “Firmicutes”, (c) “Bacteroidota”, (d) “Deferribacterota” levels. *p < 0.05, **p < 0.01. Data are expressed as mean ± SD (*n* = 3).

Aging can modify the microbial composition, such as the Firmicutes/Bacteroid ratio (Ma et al. 2021). Furthermore, the augmented abundance of Deferribacteres is linked to a pro‐inflammatory response that closely associates with aging (Berry et al. 2015). As shown in Figure 6c, at the phylum level, the top 6 prevalent microbiota were Bacteroidota, Firmicutes, Proteobacteria, Desulfobacterota, Deferribacterota, and Actinobacteriota. As shown in Figure 7a, upon administering D‐gal to the mice, a remarkable decrease was observed in the mean relative abundance of Proteobacteria, Desulfobacterota, and Actinobacteriota compared to the N group (*p* < 0.01), accompanied by a significant increase in the proportion of harmful bacteria Deferribacterota (Figure 7a,d) (*p* < 0.05). The intervention of tested beers mitigated some changes; all of the beers limited the rise in Deferribacterota (*p* < 0.01) as well as the decline in beneficial bacteria Actinobacteriota triggered by D‐gal. Furthermore, IPA was found to be able to limit the decrease in beneficial bacteria Proteobacteria (*p* < 0.001). Meanwhile, the mice treated with IPA and Stout showed a significantly lower harmful bacteria Desulfobacterota (a bacteria known for its endotoxin production) abundance compared to the M group (*p* < 0.05). It is noteworthy that the three beer types, particularly IPA, exhibited a significant decrease in Firmicutes and an increase in Bacteroidota when compared to the M group (Figure 7a,b); this result is consistent with the previous research (Hernández‐Quiroz et al. 2020). Bacteroidota has been proved to be inversely correlated with hyperlipidemia (Qiao et al. 2020). In the previous study, it was observed a significant increase in Bacteroidota in mice that were fed fewer melanoidin malts (Aljahdali et al. 2020). Firmicutes, a prominent component of the gut microbiota, have been implicated as a potential underlying mechanism contributing to obesity (Miura and Ohnishi 2014). The alleviating effect of tested beers, particularly IPA, on hyperlipidemia may be associated with the decrease in Firmicutes levels and the increase in Bacteroidota levels. The observed anti‐inflammatory effect of the tested beers, particularly IPA, may potentially be attributed to a reduction in Deferribacteres levels.

**FIGURE 8:**
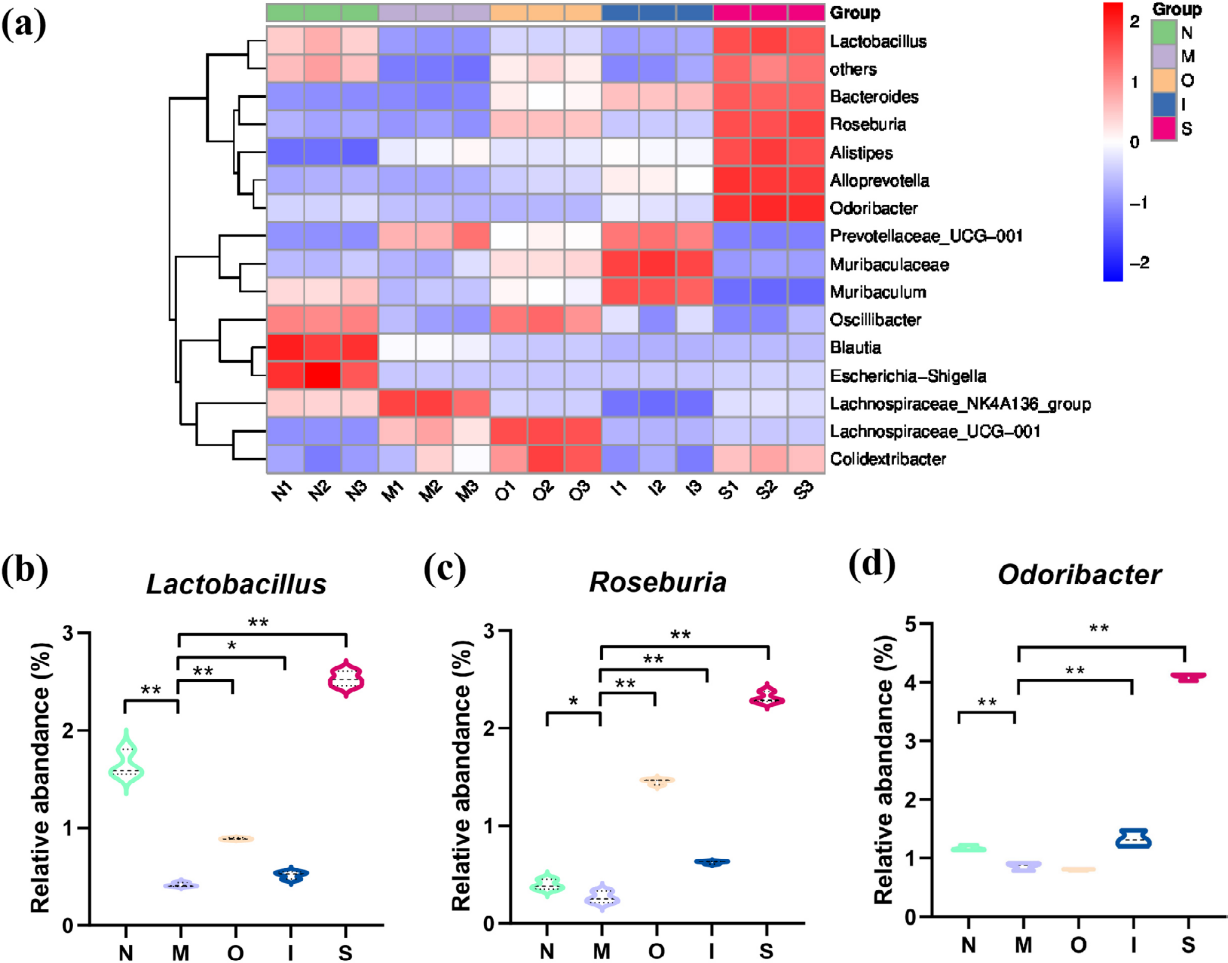
Gut microbial analysis at the genus level. (a) The heatmap analysis at the genus level; (b) *“Lactobacillus”*, (c) *“Roseburia”*, (d) *“Odoribacter”* levels. *p < 0.05, **p < 0.01. Data are expressed as mean ± SD (*n = 3*).

At the genus level, the top 15 prevalent microorganisms were shown in barplot (Figure 6d). Gram‐negative bacteria proliferate during aging and secrete lipopolysaccharide, which functions as an endotoxin and triggers inflammation in the human gut (Goto 2024). The production of short chain fatty acids (SCFAs) by the gut microbiota exerts anti‐inflammatory effects (Louis et al. 2014). However, SCFAs levels decline in elderly individuals. As shown in Figure 8a, the D‐gal‐treated mice exhibited an increased abundance of gram‐negative bacteria such as *Colidextribacter* and a decreased abundance of beneficial bacteria, including *Lactobacillus*, *Roseburia*, *Odoribacter*, *Oscillibacter*, and *Blautia*. *Lactobacillus* exerts its beneficial effects on gut flora balance through the production of a diverse array of acids (Sun et al. 2023). Previous animal studies have demonstrated the involvement of *Lactobacillus* species in modulating host lifespan (Kato et al. 2018). It has been reported that *Odoribacter* is capable of producing butyrate, which exerts a beneficial impact on gut health (Liu et al. 2020). The *Oscillibacter* genus was found to be associated with a significant reduction in both fecal and plasma cholesterol levels (Li et al. 2024). As shown in Figure 8a–d, compared with the M group, Original beer exhibited an impact on enhancing the relative abundance of beneficial bacterial genera, including *Lactobacillus*, *Roseburia, Oscillibacter*, and *Lachnospiraceae_UCG‐001*. IPA demonstrated the ability to increase the presence of the beneficial bacterial genus *Roseburia, Odoribacter*, *Oscillibacter* and decrease harmful bacteria *Colidextribacter*. Additionally, Stout administration resulted in an elevation of beneficial bacterial genera like *Lactobacillus, Roseburia, Alistipes*, and *Odoribacter*. Previous research has demonstrated the potential of microorganisms, antioxidants, fiber, and melanoidins in beer to promote the development of a healthy gut microbiota, with predominant saccharolytic, SCFAs‐producing bacteria (Zugravu et al. 2023). Ferulic acid is a prominent polyphenol found in beer, which has been demonstrated to enhance the microbial diversity and promote the proliferation of propionate‐ and butyrate‐producing bacteria in the colon of rats (Takagaki and Nanjo 2015). The antibacterial role of melanoidins has been reported, as they effectively inhibit the growth of pathogenic species (Langner and Rzeski 2014). In conclusion, several functional constituents present in the tested beers may contribute to their beneficial effects on the intestinal microbiota.

### 3.8 Changes in Community Composition of the Intestinal Microbiota in Mice

To identify the gut microbiota exhibiting the most significant differences resulting from various beer interventions, we conducted LEfSe analysis on the microbial community, as depicted in Figure 6a,b. The normal group was dominated at the genus level by *Blautia, Escherichia‐Shigella, Sporosarcina, Paenalcaligenes*, and *Acinetobacter*. The D‐gal aging model group was dominated by *Lachnospiraceae_NK4A136_group*. *Lachnospiraceae_UCG‐001* is the dominant bacterium in the O group. A previous study has reported that *Lachnospiraceae_UCG‐001* is an anti‐inflammation bacterium for its ability to produce SCFAs; moreover, it has been proven to have a negative correlation with high blood lipids in type 2 diabetes (Wei et al. 2018). The IPA‐treated group was dominated by *Muribaculaceae*, *Prevotellaceae_UCG‐001*, *Muribaculum*, *Coriobacteriaceae_UCG_002*, and *Parasutterella*. It was reported that *Muribaculaceae* could provide the foundation for ameliorating inflammation and metabolic diseases (Geng et al. 2024). *Coriobacteriaceae_UCG_002* was considered to be a beneficial bacterium for improving immune status, and it was found to be decreased in mice with liver fibrosis (Zhang, Lu, et al. 2023). The Stout‐treated group was dominated by beneficial bacteria such as *Odoribacter, Lactobacillus*, *and Roseburia. Roseburia* has been proven to be positively correlated with the fecal SCFAs, and its prevalence was significantly higher among centenarians (Wang et al. 2015).

### 3.9 Gut Microbial Interactions of Relevance for Aging in Mice

**FIGURE 9:**
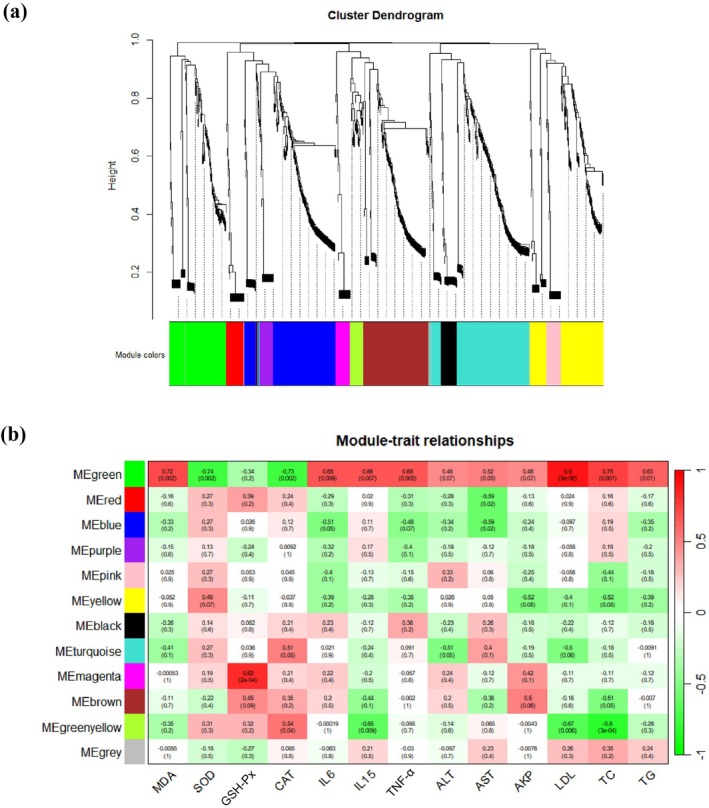
*WGCN*A analysis of gut microbes at the genus level. (a) Different modules that were classified from gut microbes; (b) Correlations between aging‐related indexes and modules in the microbial network. The heatmap illustrates the module‐trait relationship. Each row represents different modules, and each column represents aging‐related indicators.

**FIGURE 10:**
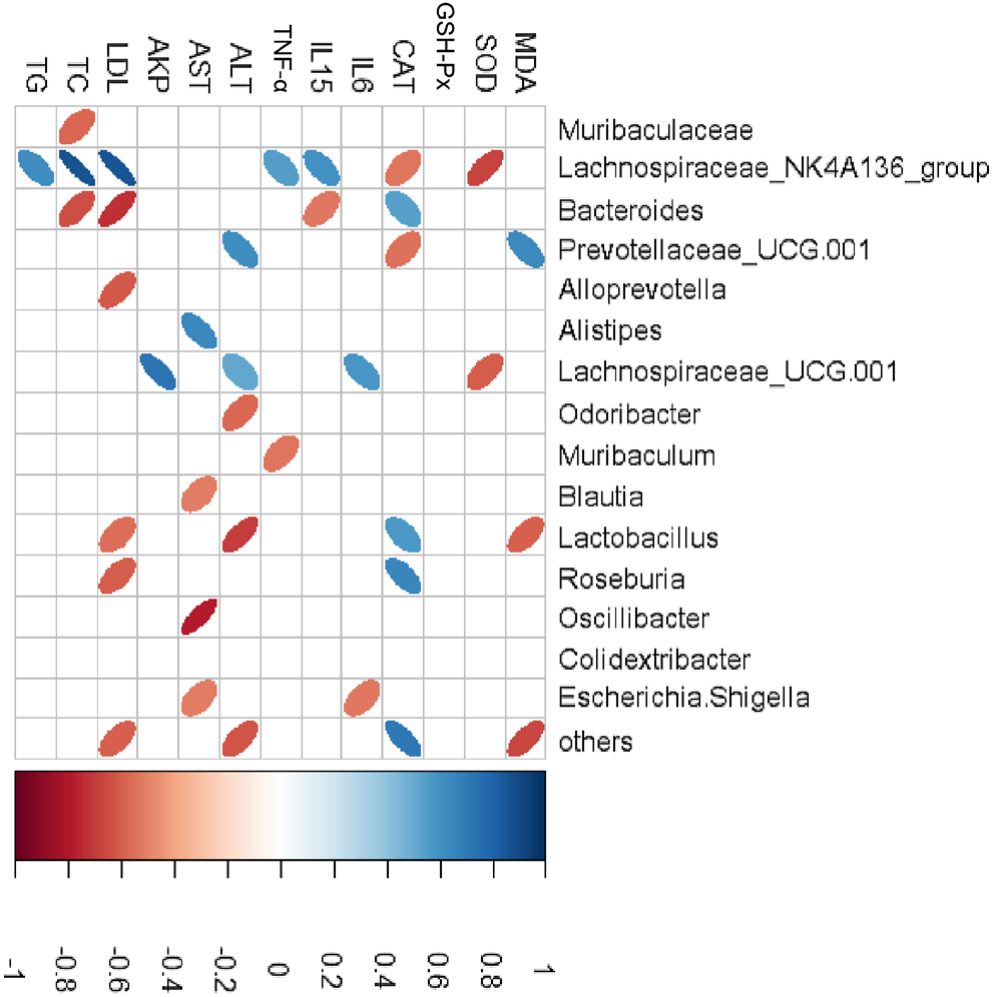
Spearman's correlation analysis of the genera and aging‐related indicators. The red ellipses depict negative correlations, while the blue ellipses represent positive correlations. The intensity of color corresponds to the strength of correlation.

The correlation between metabolic traits and modules was analyzed using WGCNA (Langfelder and Horvath 2008). Consequently, a total of 12 distinct modules were identified as independent entities (Figure 9a). As illustrated in Figures 9b and 10 modules were obviously associated with aging‐related metabolic traits. For example, the green module showed strong positive correlations with inflammation, hepatic, and serum lipid‐related indicators, and showed negative correlations with SOD and CAT (correlation coefficient = −0.74 and −0.73, respectively, *p* < 0.05). The blue module was negatively related to serum IL‐6 and AST (*p* < 0.05). The yellow module was negatively related to serum AKP and TC (*p* < 0.05). The turquoise module was positively correlated with CAT and negatively related to ALT (*p* < 0.05). The green‐yellow module showed a positive correlation with CAT (*p* < 0.05) and negative correlations with IL‐15, LDL, and TC (*p* < 0.01). As depicted in Figure S1, the majority of bacteria within the green module are classified as Firmicutes (65%), with approximately 48% of these bacteria belonging to *Lachnospiraceae*. In conclusion, WGCNA revealed a strong association between the interactions among gut microbes and aging‐related indicators in D‐gal‐treated mice. The top 10 core genera in this network were extracted with the maximum neighbor component (MNC) in the cytoHubba plugin (Figure S2). According to their high centrality, these genera can greatly influence the network structure of the microecological interactions.

A person‐correlation test was performed subsequently, as shown in Figure 10. The correlation analysis revealed that *Lachnospiraceae_NK4A136_group, Bacteroides, Prevotellaceae_UCG‐001, Lachnospiraceae_UCG‐001*, and *Lactobacillus* showed strong correlation with the indicators. *Lachnospiraceae_NK4A136_group* was positively related to IL‐15 (*p* < 0.05), TNF‐α (*p* < 0.05), LDL (*p* < 0.01), TC (*p* < 0.01) and TG (*p* < 0.05), while showing a negative relationship to serum SOD (*p* < 0.01) and CAT (*p* < 0.05). *Bacteroides* was negatively associated with IL‐15 (*p* < 0.05), LDL (*p* < 0.01) and TC (*p* < 0.01), while positively related to CAT (*p* < 0.05). *Lachnospiraceae_UCG‐001* was found to be positively related to IL‐6 (*p* < 0.05), ALT (*p* < 0.05) and AKP (*p* < 0.01), while showing a negative relationship to SOD (*p* < 0.05). *Prevotellaceae_UCG‐001* was positively related to MDA, ALT (*p* < 0.05) and negatively associated with CAT (*p* < 0.05). *Lactobacillus* was positively related to CAT (*p* < 0.05) and negatively related to MDA (*p* < 0.05), ALT (*p* < 0.01) and LDL (*p* < 0.05). In conclusion, the tested beers, especially Stout, demonstrated an increase in *Bacteroides* and *Lactobacillus* levels, while concurrently reducing the levels of *Lachnospiraceae_NK4A136_group* and *Prevotellaceae_UCG‐001*, thereby ameliorating metabolic disorders induced by D‐gal.

### 3.10 Intestinal Microbiota Function in Mice

**FIGURE 11:**
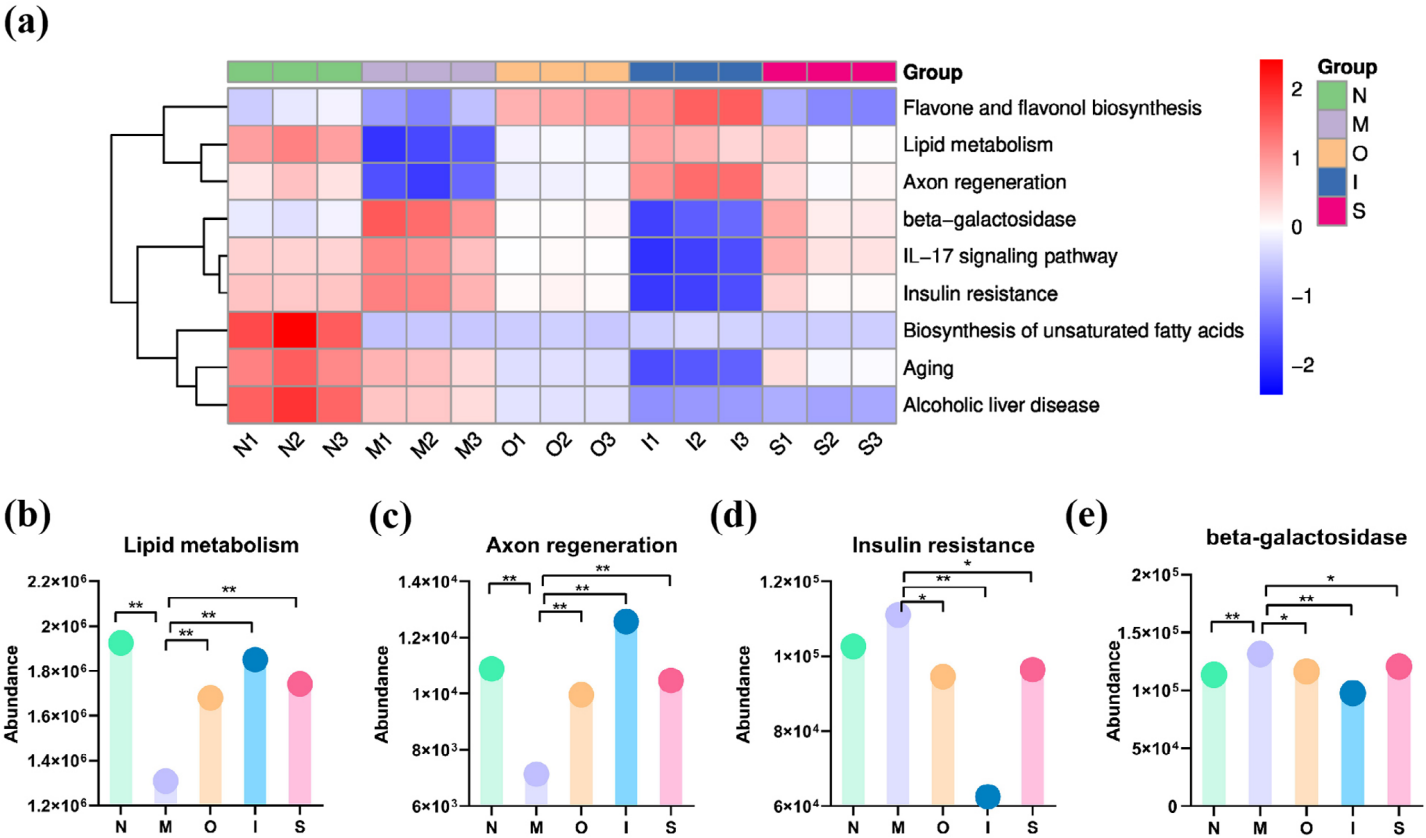
The function of gut microbiota in D‐gal‐induced aging mice. (a) KEGG difference results on a clustering heatmap; (b) “Lipid metabolism”, (c) “Axon regeneration”, (d) “Insulin resistance” and (e) “beta‐galactosidase” relative abundance. *p < 0.05, **p < 0.01. Data are expressed as mean ± SD (*n* = 3).

Moreover, KEGG enrichment analysis unveiled altered pathways associated with the process of aging across these groups (Figure 11a–e). It suggested that the changes in gut microbiota caused by D‐gal may obviously induce some changes including the decreased “Lipid metabolism”, “Biosynthesis of unsaturated fatty acids”, “Axon regeneration”, and “Flavone and flavonol biosynthesis”, as well as the increased “IL‐17 signaling pathway”, “Insulin resistance”, “beta‐galactosidase”. Compared with M group, three tested beers induced higher abundances of pathways including “Lipid metabolism” and “Axon regeneration”, while showing lower abundance of “IL‐17 signaling pathway”, “Insulin resistance”, “beta‐galactosidase” functions. Epidemiological studies showed that moderate beer consumption reduces the risks of cardiovascular and neurodegenerative diseases (de Gaetano et al. 2016). Notably, “aging” was significantly down‐regulated in beer treatment groups compared with N and M groups. The results revealed that tested beers had some beneficial changes in aging‐related pathways.

## 4 Conclusion

In this study, we conducted a comparative analysis of the compositional differences among Original beer, IPA, and Stout. By employing a D‐gal‐induced aging mice, we demonstrated that moderate consumption of tested beers effectively alleviated the detrimental effects induced by D‐gal administration. Notably, IPA and Stout exhibited more pronounced improvements, potentially attributed to their high levels of polyphenols and analogous compounds. Additionally, the tested beers exhibited the ability to restore microbial diversity in colonic contents; the Original beer demonstrated a more pronounced improvement compared to IPA and Stout, potentially attributed to its higher abundance of *Saccharomyces cerevisiae* strain. Moreover, the tested beers elicited modifications in the composition of intestinal microbiota, including a reduction in pathogenic bacteria such as Deferribacterota and an augmentation of beneficial bacteria like *Lactobacillus* and *Roseburia*, accompanied by a decline in Firmicutes abundance and an elevation in Bacteroidota levels. The alteration of intestinal flora may lead to changes in metabolite levels, thereby influencing the metabolic processes of intestinal cells. This, in turn, modulates oxidative stress, inflammatory senescence, and hyperlipidemia, thereby retarding the aging process. In conclusion, our study provides valuable insights into the favorable impacts associated with moderate beer consumption on human aging and health. We recommend that research related to the consumption of alcoholic beverages should be carried out with moderate consumption and specify the specific type of alcoholic beverage used, so as to standardize the results found and enable comparison between studies. Our future investigations should focus on identifying the effector molecules and signaling pathways underlying their beneficial effects.

## Author Contributions

**Xueyuan Fu:** data curation (lead), writing – original draft (lead), writing – review and editing (lead). **Changwei Wang:** investigation (lead), project administration (lead). **Zhaoxia Yang:** conceptualization (equal), methodology (equal). **Junhong Yu:** conceptualization (equal), investigation (equal). **Jianfeng Wang:** methodology (equal), supervision (equal). **Wanxiu Cao:** formal analysis (equal), software (equal). **Chuyi Liu:** investigation (equal), validation (equal). **Hua Yin:** project administration (equal), supervision (equal). **Bafang Li:** conceptualization (equal), supervision (equal). **Xiaomei Feng:** resources (equal), visualization (equal). **Fen Du:** visualization (equal). **Hu Hou:** funding acquisition (equal).

## Conflicts of Interest

The authors declare no conflicts of interest.

## Supporting information


**Figure S1.** Network of the gut microbes in each module identified by WGCNA. Different colors represent the different modules.
**Figure S2.** Identification of core genera in the microecological co‐expression network using cytoHubba plugin. The rank of the connection degree is represented by different colors (from red to yellow).
**Figure S3.** Graphical representation of effect of the tested beers on D‐gal‐induced aging mice.

## Data Availability

The authors have nothing to report.
