# Supplementary material for: Moderate Beer Consumption Ameliorated Aging‐Related Metabolic Disorders Induced by D‐Galactose in Mice via Modulating Gut Microbiota Dysbiosis
Source: Food Sci Nutr. 2025 Aug 11;13(8):e70678. doi: 10.1002/fsn3.70678 (PMC12339418; doi:10.1002/fsn3.70678)
Supplement: Supplementary file 1 — Figure S1. Network of the gut microbes in each module identified by WGCNA. Different colors represent the different modules. Figure S2. Identification of core genera in the microecological co‐expression network using cytoHubba plugin. The rank of the connection degree is represented by different colors (from red to yellow). Figure S3. Graphical representation of effect of the tested beers on D‐gal‐induced aging mice. [file FSN3-13-e70678-s001.docx]

**Appendix: Supplementary information**

**
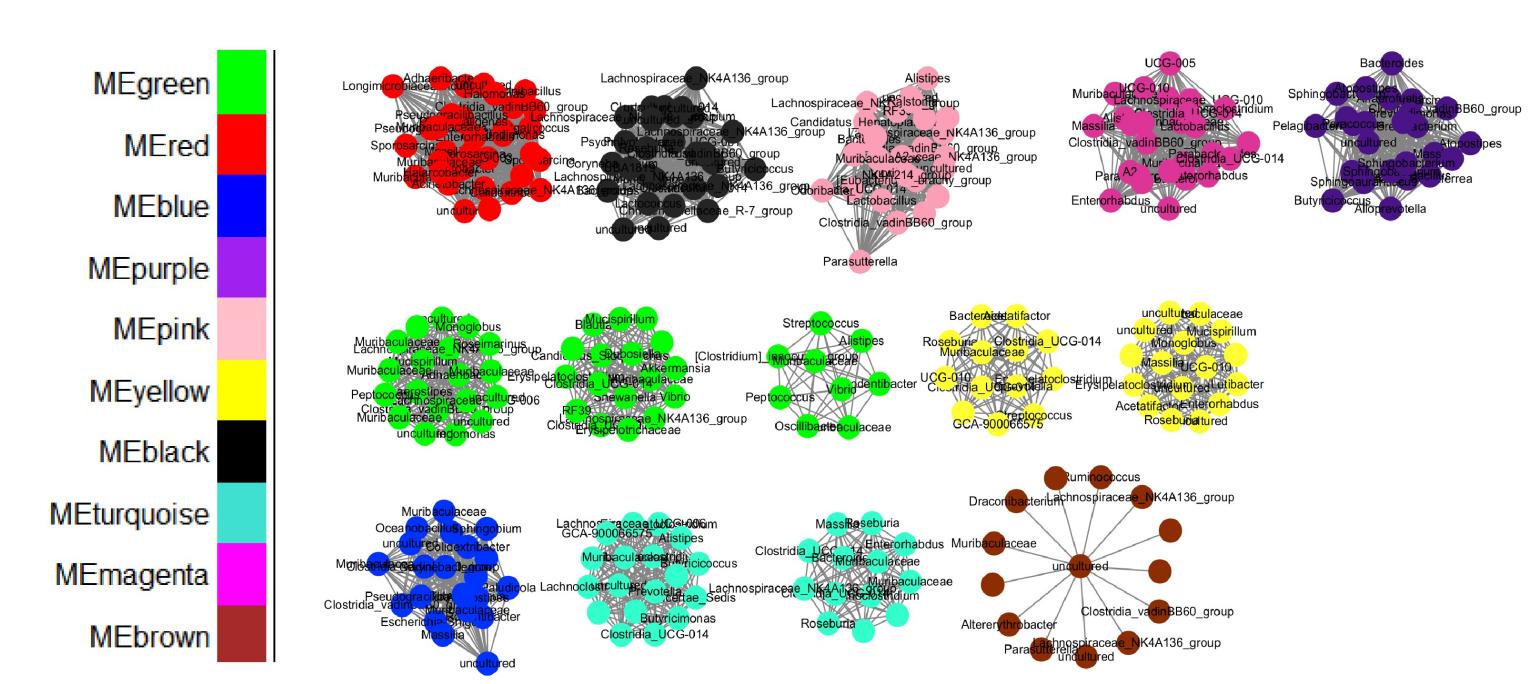
Supplementary FIGURE S1.** Network of the gut microbes in each module identified by WGCNA. Different colors represent the different modules.

**Supplementary FIGURE S2**. Identification of core genera in the microecological co-expression network using cytoHubba plugin. The rank of the connection degree


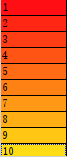

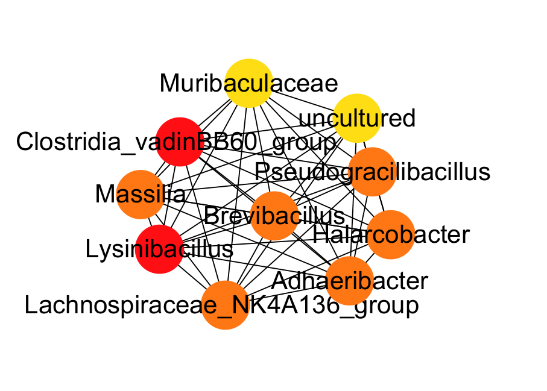


is represented by different colors (from red to yellow).

**
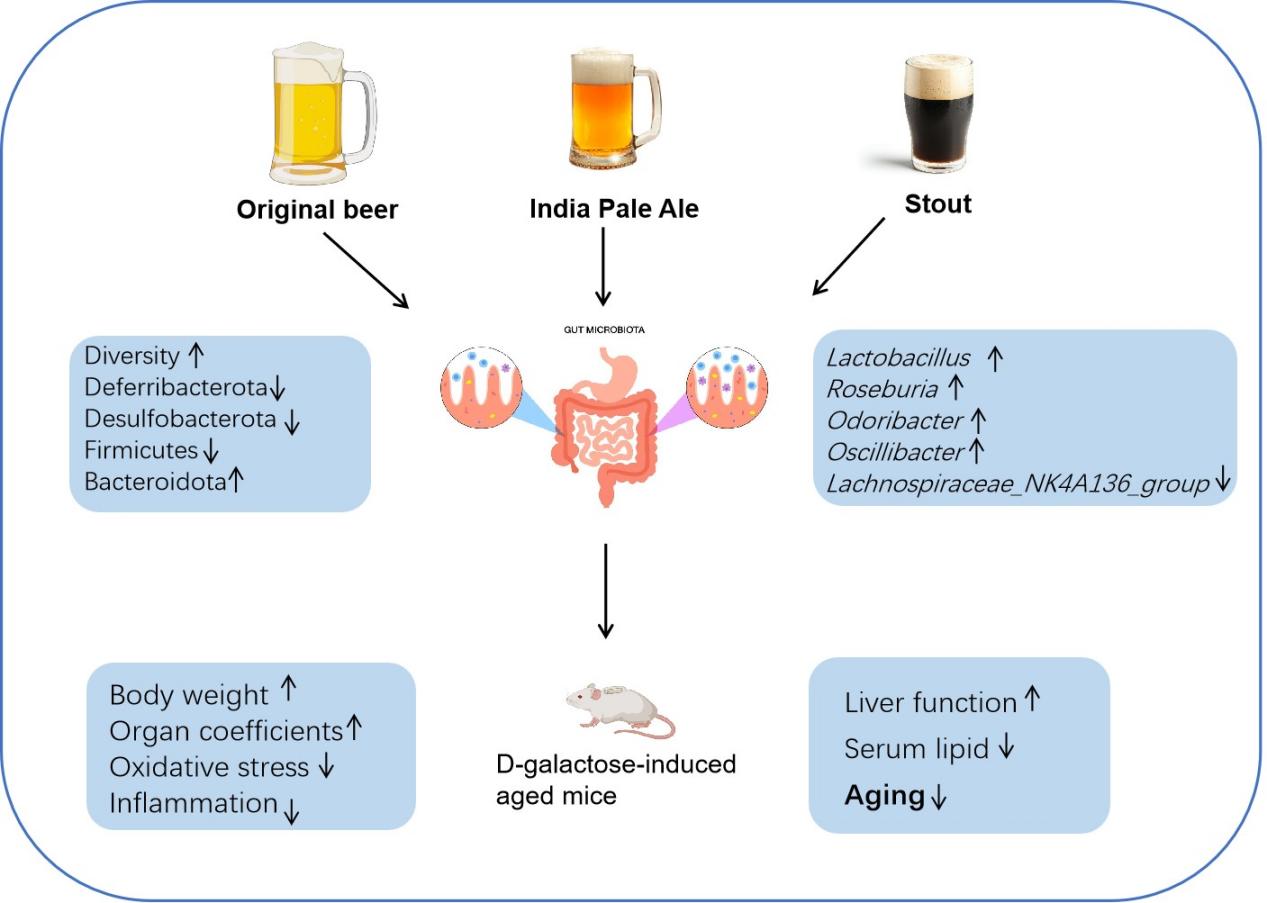
Supplementary FIGURE S3**. Graphical representation of effect of the tested beers on D-gal-induced aging mice.
